# Stability evaluation and validation of appropriate reference genes for real-time PCR expression analysis of immune genes in the rohu (*Labeo rohita*) skin following argulosis

**DOI:** 10.1038/s41598-023-29325-1

**Published:** 2023-02-15

**Authors:** Pramoda Kumar Sahoo, Sonali Parida, Subhadarshini Parida, Priyashree Parida, Anirban Paul

**Affiliations:** National Referral Laboratory for Freshwater Fish Diseases, Fish Health Management Division, ICAR-Central Institute of Freshwater Aquaculture (CIFA), Kausalyaganga, Bhubaneswar, 751002 India

**Keywords:** Biotechnology, Molecular biology

## Abstract

Argulosis is one of the most unrestrained economically significant freshwater fish ectoparasitic diseases. Proper selection or normalization of the best reference gene governs the accuracy of results of gene expression studies using real-time PCR. Earlier studies in rohu carp (*Labeo rohita*) have used reference genes without proper validation. Here, seven candidate reference genes viz., acidic ribosomal protein (ARP0), glyceraldehyde 3-phosphate dehydrogenase, RNA polymerase II (RPo), elongation factor1α (EF1α), α- tubulin (AT), ribosomal protein L 10, and β-actin were evaluated using four algorithms (geNorm, BestKeeper, NormFinder and ∆Ct) followed by a comprehensive gene expression analysis using skin tissue of rohu at varied time points of experimental *Argulus siamensis* infection. ARP0 and EF1α were found to be the most stable whereas RPo and AT were considered as least stable genes based on basal expression level and variation in expression levels. Validation of candidate reference genes was undertaken by looking into the expression of six immune-related genes using the two most stable and two least stable genes as housekeeping genes in *Argulus*-infected rohu skin at different time points of infection. An increased expression of immune genes indicated the role of inflammation and the immune modulation process at the site of attachment of parasites in governing infection.

## Introduction

Rohu carp (*Labeo rohita*) is the most preferred and cultured fish species among three Indian major carps that contributes to 3.7% to the global finfish aquaculture production^1^. Different parasites are found to be the most predominant etiological agents in Indian major carp farming with an overwhelming incidence rate of 74.88% followed by bacterial, viral and fungal infections^2^. Among different parasitic infections, *Argulus* is the most common ectoparasite in the carp culture system^2^ and also one of the most economically important parasites of finfish in the globe^3^. The parasite punctures the fish skin, injects toxin (cytotoxic) through oral sting and feeds on blood, epithelial cells and mucus^4^ leading to hemorrhage and ulcer at the feeding site, thus giving access to opportunistic pathogens. More specifically, infection with *Argulus siamensis* has appeared as an important drawback in polyculture ponds in India^5,6^. This parasite causes a significant economic loss to Indian carp farming amounting to INR 3000 million (~ 625,000 US$) per annum^7,8^. The current methods of control using anti-parasitic drugs (avermectins, molt-inhibitors, synthetic pyrethroids, organophosphates, etc.) are not only leading to drug resistance in parasites but also have off-target effects^8^. Hence, understanding host–pathogen interaction, particularly through looking into the expression of immune-related genes may give some leads towards developing immunoprophylaxis against this pathogen for long-term protection. Although our earlier studies have looked into expression analysis of immune-related genes using this parasite-fish model^9,10^ besides few studies in other fish species^11–13^, in most of the cases β-actin has been used as reference gene without proper validation.

Measurement of modulation of gene expression is an indispensable research approach to comprehend and divulge the complex regulatory gene network in animals^14^ and play a major role in understanding host immune regulation against disease progression. There are many methods available to measure the expression levels of the gene of interest. Among them, real-time quantitative polymerase chain reaction (RT-qPCR) is a universally adopted strategy for computing gene expression modulation due to its specificity, sensitivity, reproducibility and convenience. This technique is also in wide use in gene expression analysis, miRNA analysis, copy number variation (CNV) analysis, single nucleotide polymorphism (SNP) genotyping, specificity of product through melt curve analysis, etc. Despite these advantages, normalization to a constitutively expressed gene in RT-qPCR is required to elude experimental errors caused by sample variations, disparities in quality and integrity of RNA, enzymatic efficiency for cDNA transcription and PCR efficiency. However, the transcript levels of genes vary across cellular conditions, which indicates one reference gene should not be routinely used as reference genes in all gene expression studies. The ideal reference gene for qPCR should have a constant level of expression in various tissues/cells or developmental stages and should be unaffected by experimental situations^15^. Many research reports present gene expression results using reference genes without appropriate validation of their stable expression. The gene transcription in fish is very intricate process due to high individual variation and multiple isoforms and subtypes of genes^16^. On the other hand many research papers describe target gene transcript modulation following infection, only a limited number of articles exhibit validation of reference gene in specific tissue during an infection process in fish^17,18^. Further, it is essential to standardize reference genes for each infecting pathogen for an accurate representation of undergoing immune gene regulation during infection. As it is essential to understand and evaluate gene expression during *Argulus* infection, which infects mostly skin tissue, the current study was undertaken to select, screen and validate candidate reference genes for RT-qPCR study in rohu (*Labeo rohita*) skin tissue during *A. siamensis* infection.

## Materials and methods

### Experimental fish

Apparently healthy *Labeo rohita* (rohu) fingerlings of size 25–35 g were received from the ICAR-Central Institute of Freshwater Aquaculture fish farm and maintained in the wet laboratory. Representative fish samples were screened for the presence of any pathogens using level I and level II diagnosis. Disease free fishes were kept for a period of 20 days for acclimatization at 25–28 °C under continuous aeration. The fishes were fed with commercial pellet feed at 3% of body weight. Optimum water quality was maintained and the water exchange was undertaken at 10–20% every day for the removal of uneaten feed and other faecal matters. All the experiments were performed following the approval of the Institute Animal Ethics Committee (ICAR-CIFA/IAEC/14.02.2022). Further, all study protocols were performed according to the Explanation and Elaboration accordance to the ARRIVE guidelines 2.0. All methods were performed following relevant guidelines and regulations.

### Parasite collection and maintenance

A population of *A. siamensis* was maintained on stock *Labeo rohita* (approximately 500 g) in a separate tank. Parasite eggs collected from the tanks were used to produce metanauplii in the laboratory using previously standardized protocol^19^. The hatched out metanauplii were later used for challenge experiments in experimental fishes using metanauplii for creating infection within 6–8 h of hatching.

### Experimental design

A total 21 numbers of acclimatized *Labeo rohita* were placed in 500 L capacity FRP tank. The challenge experiment was carried out by exposing 100 numbers of metanauplii per fish following Kar et al.^9^. The experiment was conducted for 30 days. At each time point i.e. 0, 1, 5, 10, 20, 30 days’ post-infection (dpi), 3 fishes were randomly sampled and skin tissue was collected in RNALater™ (Sigma-Aldrich, USA) and stored at − 20 °C for RNA isolation and subsequent analysis. Fish samples with no parasite on body from the acclimatized tank at day 0 served as control. At each time point fishes were checked for parasite presence in the fish body to confirm infection.

### Selection of reference gene

Based on former studies^20,21^ seven reference genes viz., acidic ribosomal protein (ARP0), glyceraldehyde 3-phosphate dehydrogenase (GAPDH), RNA polymerase II (RPo), elongation factor1α (EF1α), α- tubulin (AT), ribosomal protein L 10 (RL 10), and β-actin were selected as candidate reference genes in the present study. The details of selected candidate reference genes are mentioned in Table 1. Primers for all the candidate genes except β-actin were self-designed using Primer Premier 5 (version 5.0, Premier Biosoft International, Palo Alto, CA) from the sequences obtained from the available rohu transcriptomics data in our laboratory (Table 2). The primer specificity was checked for all selected genes using semi-quantitative PCR followed by amplification of desired size of the amplicons and commercial sequencing (AgriGenome, Kochi, India).

**Table 1:** List of reference genes used in this study.

Sl. no	Abbreviation	Full name	Function
1.	ARP0	Acidic ribosomal protein	Protein synthesis
2.	GAPDH	Glyceraldehyde 3-phosphate dehydrogenase	Enzyme involved in glycolysis
3.	RPo	RNA polymerase II	Synthesis of precursor mRNA
4.	EF1α	Elongation factor 1α	Protein synthesis
5.	AT	α-Tubulin	Cytoskeletal protein
6.	RL10	Ribosomal protein L 10	Ribosome biogenesis and ribosome function
7.	β-ACTIN	β-Actin	Cytoskeletal protein

**Table 2:** Reference gene primers used in this study.

Sl. No	Name	Sequence (5′ to 3′)	Correlation (R^2^)	Amplification coefficient (E)	Reference
1.	ARP0	F-CCTGCACAAGAGATTCCT R-GTTGATGACGGAGTGAGG	99	91.18	Self-designed
2.	GAPDH	F-AACTCACCAAGTTTTGCGACAG R-AGGTGGGAACAGGAATGCTAAG	98	101.8	Self-designed
3.	RPo	F-GACAAGAGGACATGCCATT R-GTGAAGCCATTATACAGAACC	99	116.22	Self-designed
4.	EF1α	F-TTTGCTGTGCGTGACATGAG R-GGGTGTCTGACGGGATGATT	99	97.32	Self-designed
5.	AT	F-TGATGTACGCCAAGAGAGCTT R-TTCATCCTCTTCTCCGACGC	98	109.29	Self-designed
6.	β-actin	F-TGGCAATGAGAGGTTCAGGT R-TGGCATACAGGTCCTTACGG	97	117.89	^9^
7.	RL10	F-AAGCGTTTCTCAGGCACTGT R-TGTCCATGTGTGGAAGCTCA	95	103.81	Self-designed

### RNA isolation and cDNA synthesis

The skin samples collected at different time intervals from *L. rohita* were subjected to RNA extraction using TRIzol reagent (Sigma-Aldrich, St. Louis, MO, USA) according to the manufacturer’s instruction followed by DNase I (Fermentas, Thermo Fisher Scientific, Wilmington, DE, USA) treatment and inactivation of DNase I. The purity of RNA was checked by measuring the ratio of OD260 nm and OD280 nm using NanoDrop ND1000 (Thermo Scientific) followed by running in 1% agarose gel and also looking into the expression of β-actin using semi-quantitative PCR as detailed later^22^. RevertAid First Strand cDNA synthesis kit (Thermo Fischer Scientific Inc., USA) was used to generate complimentary DNA from 1 μg of total RNA by reverse transcription following the manufacturer's instruction. The synthesized cDNA was stored at − 20 °C until used.

### Quantitative real-time PCR (RT-qPCR)

Skin tissue cDNA of different time periods were used as amplicon to perform RT-qPCR using a LightCycler 96 SW 1.1 (Roche, Germany) as described earlier^23^. Each sample comprises of three biological replicates and two technical replicates. No amplicon negative control was included in each run. The pooled cDNA were ten-fold serially diluted to analyse the slope of the standard curve for each gene for calculating PCR efficiency (E) and correlation coefficient (R^2^) using formula E (%) = {(10^−1/slope^−1) × 100}^24–26^.

### Stability and quantitative analysis of reference genes

**Figure 1:**
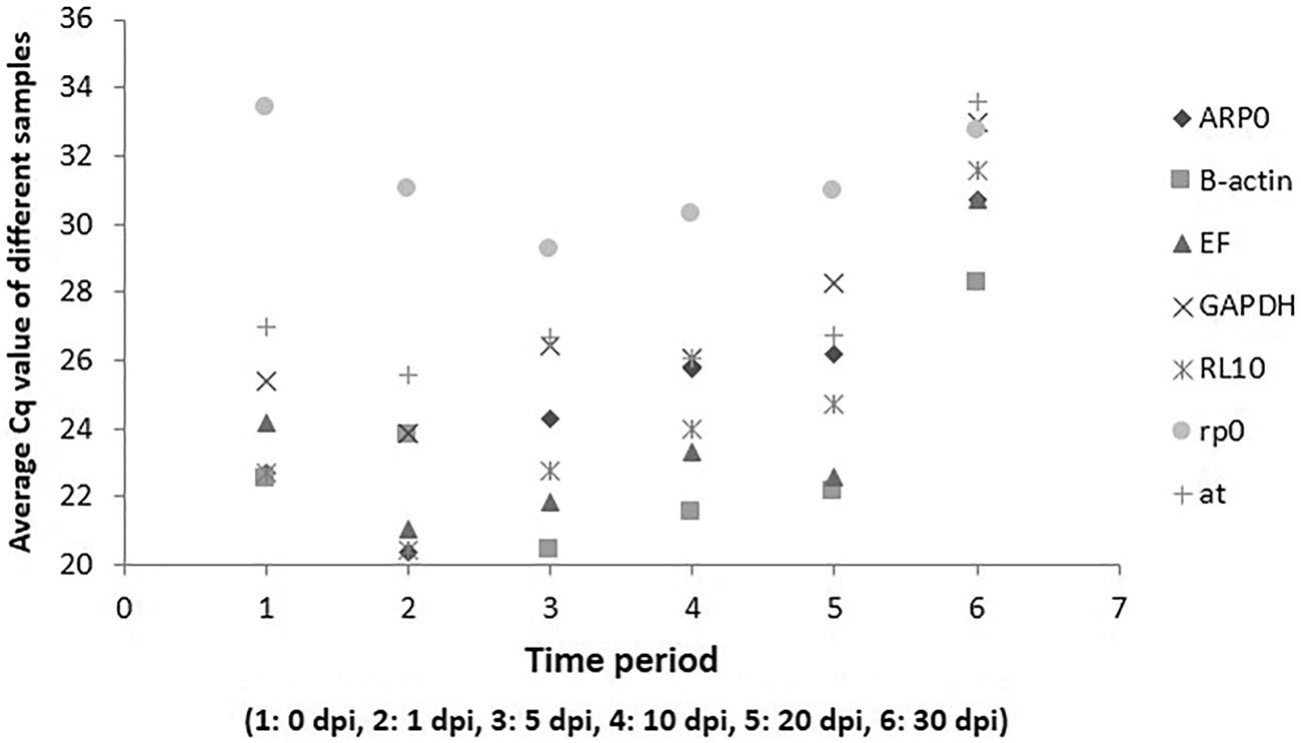
RNA transcription levels of candidate reference genes (absolute Cq values) representing the abundance of studied genes in each time period.

The abundance of selected candidate reference genes within different skin samples were analysed by a direct comparison of Cq (quantification cycle) values (Fig. 1). The most stable reference gene among seven selected reference genes was evaluated using four extensively used algorithms i.e. comparative ΔCt, geNorm, NormFinder and BestKeeper^27–29^. The expression level of each gene was determined from the cycle threshold (Cq values) in all algorithms. These four algorithms together produced a comprehensive ranking of the most stable gene among the candidate genes.

### Validation and application of stable reference gene(s) for expression analysis of immune genes in *L. rohita* skin against *A. siamensis* infection

Comparative fold change of different selected innate and specific immune genes mentioned in Table 3 were analyzed by using two most stable reference genes (ARP0 and EF1α) and two least stable reference genes (RPo and AT) of *L. rohita* following Sahoo et al.^8^.

**Table 3:** Primer details of selected immune genes of *L. rohita.*

Sl. No	Immune genes	Sequence (5′-3′)	Ta (°C)	References
1.	Major histocompatibility complex I (MHC I)	F-CAGTATGGGTATGATGGA R-TCTGCCAGGAGATTGTT	54	^41^
2.	Major histocompatibility complex II (MHC II)	F-AGGAGATGCCGAATGGAG R-GATGATTCCCAGCACCAG	55.3	^41^
3.	Immunoglobulin D (IgD)	F-GGTTGACTCTTAAGACCGTTGT R-GGGTCCATCCACACAACTTA	52.1	^9^
4.	Toll-like receptor 22 (TLR22)	F-TCACCCCATTTCGAGGCTAACAT R-GAAGGCGTCGTACTGGAATGTC	56	^41^
5.	Interleukin-10 (IL-10)	F-CTCATTTGTGGAGGGCTTTC R-ATGCCAGATACTGCTCGATG	52	^41^
6.	Apolipoprotein A-I (ApoA-I)	F-TGGAGGCTGTGCGTGTA R- GCTCGCCCAGTTCATTC	59	^41^

The quantification cycle (Cq) values were calculated using the comparative Cq method (in triplicate). The Cq value of the individual gene for each cDNA was subtracted from its respective Cq value of different reference genes individually to get the ΔCq value. Further, by subtracting the average ΔCq of each of the samples from the average ΔCq value of the calibrator, ΔΔCq was calculated. Fold difference for each gene against each reference gene was calculated as 2^−ΔΔCq^. The mean fold difference was calculated and represented as ± standard error.

### Statistical analysis

The difference between the mean values of the gene at a particular time point was compared to its respective control using Student’s t-test. The differences in the expression levels of various immune genes were represented as mean ± SE and analyzed using one-way ANOVA followed by Duncan’s multiple range tests (significance level as p < 0.05).

## Results

### Primer specificity, amplification efficiency and transcription level of candidate reference genes

PCR product of expected amplicon size for each reference gene primer was visualized in 1.5% agarose gel electrophoresis and the amplicons were confirmed by sequencing and blast analysis. Melt curve analysis with a single peak for each candidate reference gene further confirmed the amplification specificity (Suppl. Figure 1a–g). The calculated PCR efficiencies from the standard curve generated using tenfold serial dilution of pooled cDNA for seven selected different genes were varied from 91 to 118% (Table 2). The expression level of candidate reference genes was determined by RT-qPCR at different time intervals post-*Argulus* infection. The candidate reference genes exhibited wide range of accumulation level across different time post-infection in skin samples, with threshold cycle (Cq) values ranging 19.71–38.22. The lowest expressed two reference genes with highest Cq values obtained were RPo (mean Cq = 33.41, SE = 0.423379) and AT (mean Cq = 26.995, SE = 1.304626), while ARP0 and EF1α were two of the highest expressed genes (mean Cq = 22.39, SE = 0.082669 and Cq = 24.18, SE = 2.674262, respectively) in skin of *L. rohita* during argulosis (Fig. 1).

### Expression stability of candidate reference genes

**Figure 2:**
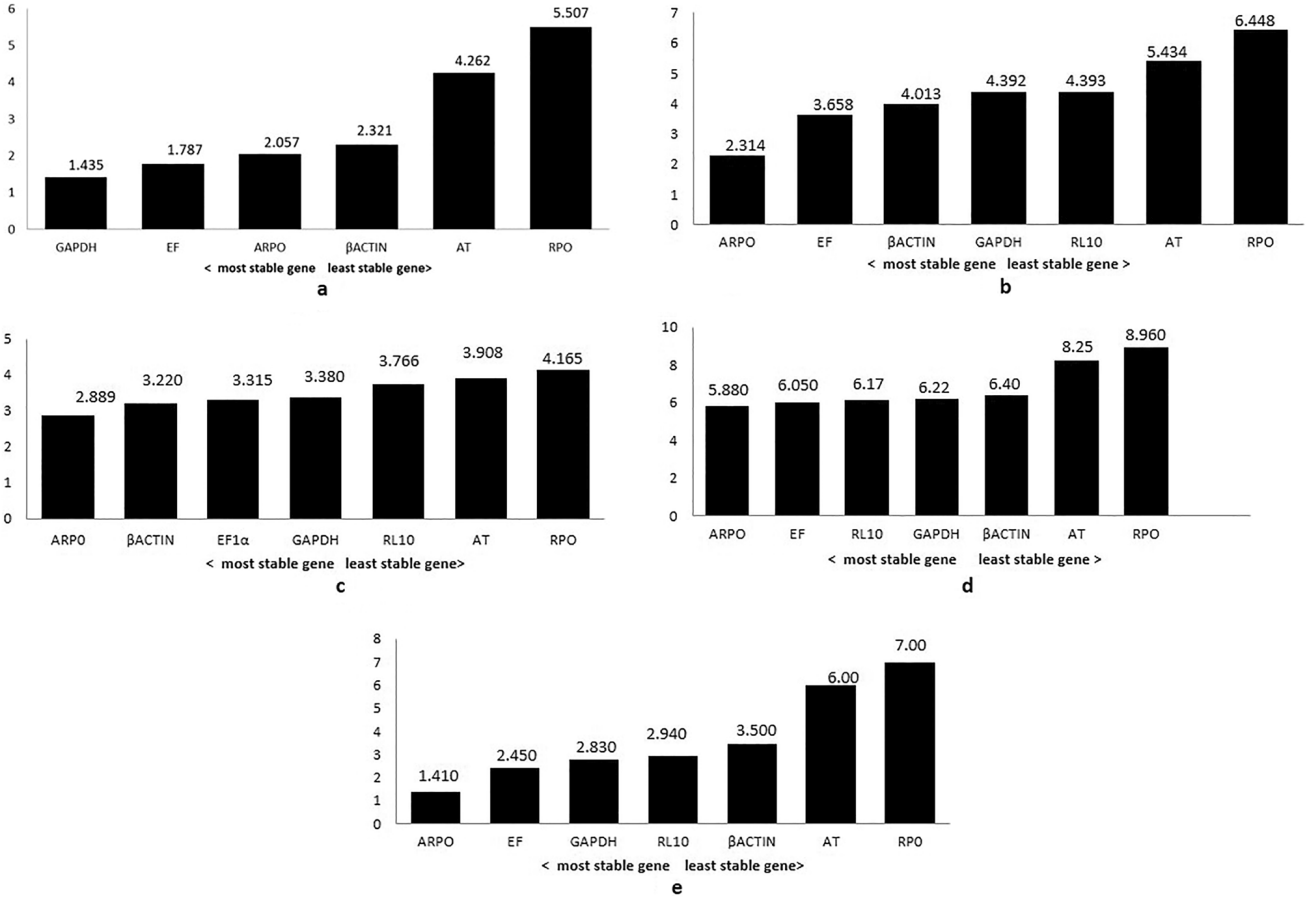
Stability value of seven candidate reference genes calculated by (**a**) geNorm, (**b**) NormFinder, (**c**) BestKeeper, (**d**) ∆Ct method and (**e**) comprehensive ranking in different post infection periods.

The expression stability of candidate reference genes at different time intervals during *A. siamensis* infection was determined using four algorithms (geNorm, NormFinder, BestKeeper and ΔCt). geNorm analysis ranked GAPDH/RL10 and EF1α as the most stable genes, and RPo and AT as the most unstable genes (Fig. 2a; Suppl. Figure 2) among the seven candidate reference genes studied. NormFinder analysis revealed ARP0, EF1α as most stable whereas, AT and RPo as least stable genes (Fig. 2b). BestKeeper algorithm determined two most stable genes were ARP0 and β-actin and the other genes in the rank from most stable to unstable were observed as EF1α, GAPDH, RL10, AT and RPo (Fig. 2c). Comparative ΔCt method algorithm found ARP0 and EF1α as the most stable genes while RPo and AT as the most unstable genes (Fig. 2d) (Suppl. Tables 1a, b).

Further, by combining these four algorithms a comprehensive ranking of all seven genes was generated by an online tool i.e. RefFinder (Fig. 2e). Comprehensive ranking indicated ARP0 and EF1α as the most stable candidate reference genes for *L. rohita* skin infected with *Argulus* where as RPo and AT were the least stable genes. Further, ARP0 was noticed to be the least variable candidate gene with a coefficient of variation (CV) of 0.14–15.19% followed by EF1α (CV ranging from 2.57 to 16.03%), and RPo revealed the highest variability, with CV ranging from 0.72 to 16.42% followed by AT with CV value from 2.25 to 11.18% in different time periods post *A. siamensis* infection in *L. rohita* skin. In addition, the pair-wise variation (V) calculated by geNorm method also supported ARP0 and EF1α as most reliable reference genes for normalization, and AT and RPo as the least stable genes.

### Validation of the recommended reference genes adding to the knowledge of expression of immune genes during *Argulus* infection

**Figure 3:**
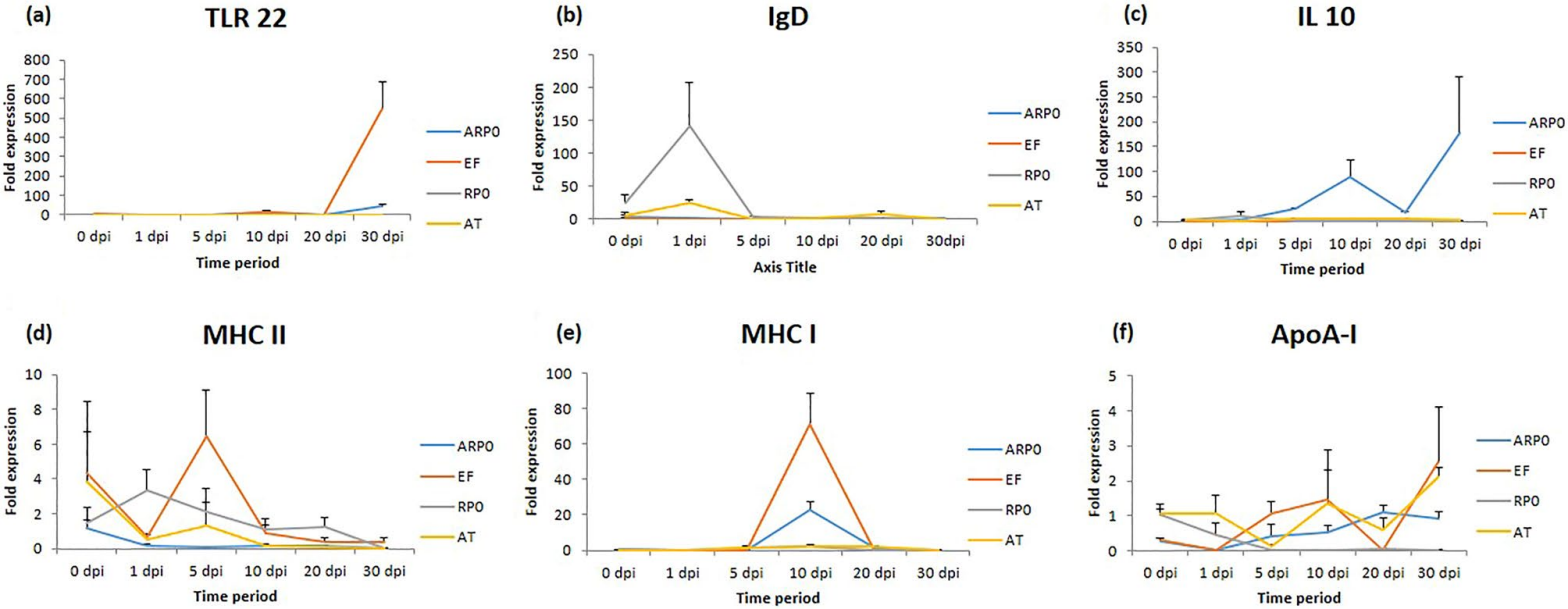
Relative gene expression of immune genes (**a**) TLR22, (**b**) IgD, (**c**) IL-10, (**d**) MHCII, (**e**) MHCI, and (**f**) ApoA-I in different post infection periods using two best stable genes (ARP0 and EF1α) and two least stable genes (RPo and AT) for normalization.

The expression of six immune-related genes has been studied in the skin tissue at different time periods post *A. siamensis* infection in *L. rohita*. The relative quantification of immune genes was carried out using identified two most stable genes i.e. ARP0 and EF1α and, RPo and AT as least stable reference genes for normalization and further validation (Fig. 3a–f). The expression of TLR22 was noticed to be unchanged up to 20 dpi. Interestingly, at 30 dpi significant up-regulation of TLR22 was observed using both ARP0 and EF1α (41.98-fold and 245.95-fold, respectively) whereas, no change observed with RPo and AT as internal control (Fig. 3a, Suppl. Table 1c). There was no change in IgD expression using housekeeping gene ARP0 during 0 h to 30 dpi, however, using EF1α, the expression was found to be down-regulated throughout the study period as compared to 0 dpi. IgD up-regulation was observed at 1 dpi when AT and RPo were used as reference genes (Fig. 3b, Suppl. Table 2). Using ARP0 as a housekeeping gene revealed up-regulation of IL-10 gene to 176.22-fold and 354.46-fold at 10 and 30 dpi, whereas, using EF1α IL-10 expression was upregulated only at 10 dpi. Interestingly, no significant change was observed in IL-10 gene expression when RPo was used as reference gene. Interestingly, using AT as reference gene IL-10 expression was found to be slightly up-regulated 5 dpi onward (Fig. 3c, Suppl. Table 3). The expression of the MHCII gene was down-regulated throughout the experimental period compared to 0 h control group and remained at a similar level up to 30 dpi when ARPo was used as a reference gene. Using EF1α and AT as reference genes, MHCII showed no change in expression in any of the time points. Using RPo as reference gene, the MHCII gene showed variable levels of expression (Fig. 3d, Suppl. Table 4). MHC I gene was not modulated throughout the experimental period using AT as reference gene. Using EF1α and ARP0 as reference genes, MHCI was found to be up-regulated to 44-fold and 357-fold at 10 dpi, respectively. Using RPo as reference gene MHCI was up-regulated at 5 dpi onward and returned back to 0 h level at 20 dpi (Fig. 3e, Suppl. Table 5). ApoA-1 was up-regulated from 10 dpi onwards using ARP0 as reference gene whereas, using EF1α no significant change was observed in any of the time point post infection. Using RPo as reference gene ApoA-I was down regulated throughout the experimental process. Further, ApoA-I was down-regulated at 5 dpi and then up-regulated 2.04 fold at 30 dpi using AT as reference gene (Fig. 3f, Suppl. Table 6).

## Discussion

Gene expression analysis is presently a very indispensable aspect for functional characterization of any gene in different species including fish. RT-qPCR is a widely used tool for gene expression studies considering its accuracy, reliability, cost-effectiveness and rapid way to analyse target genes transcript level. Recently many studies have pointed out that for obtaining precise results using RT-qPCR relies on the proper selection and normalisation of reference gene^30^ to avoid skewed results. The ideal reference gene should stably express at a constant level at different cells, tissues and even to species under different patho-physiological conditions. However, such perfect reference gene which is constitutively express in all conditions is non-existence in reality. Earlier studies indicated that there is no single gene, which is expressed stably under diverse experimental conditions^8,10,13,21,23,31^. Therefore, it is suggested to investigate most suitable housekeeping genes using different infection model to have accurate gene expression results.

In this regard, to identify best suitable reference gene of *L. rohita* during *A. siamensis* infection, seven candidate reference genes viz., ARP0, GAPDH, RNAPo2, EF1α, α-tubulin, RL10 and β-actin were selected. All the selected reference gene sequences were acquired from the transcriptome sequence data of rohu^32^ and used in this study. To evaluate the candidate reference genes four different algorithms i.e. geNorm, BestKeeper, NormFinder and ∆Ct method, were followed. Each of these algorithms has its own strength and weaknesses and there is no common consensus on which is the most reliable method. Different algorithms may generate different results depending upon the experimental variables and set of analyzed genes^33–36^. For instance, the NormFinder evaluates the intragroup variation using ANOVA and combines both variations into a stability value whereas the dCt method compare relative expression of gene pairs with SD values to derive stability index. On the other hand, geNorm calculates M-value from the SDs of gene expression and the geometric mean, and compares all gene pair’s results. BestKeeper uses also SD values and a geometric mean of the genes with the most stable Ct value to find out BestKeeper index besides calculating Pearson’s correlation coefficient and P-value^37^. The differential results obtained here by each algorithm are obvious due to use of distinct statistical approaches for the derivation of rankings^38^. Hence, these four algorithms together using RefFinder online software produced a comprehensive ranking of the most and least stable genes among the candidate reference genes which is being accepted worldwide as overall meaningful consensus^31^. In present study, comprehensive ranking has revealed ARP0 and EF1α as most stable internal controls from RefFinder analysis which were further validated in conducting immune gene expression analysis in skin tissue following ectoparasite *A. siamensis* infected *L. rohita*. Interestingly, all algorithms except geNorm resulted ARP0 as the most stable gene. It seems to be the first study to analyse and validate reference genes of rohu, *L. rohita*, an important widely cultured Indian major carp species of aquaculture system and particularly using skin tissue for gene expression analysis. Although most of the earlier gene expression studies in rohu were conducted either using β-actin or EF1α^6,9,39–41^, ARP0 has never been attempted previously. The second stable reference gene was EF1α as noticed here. Earlier studies in different fish species also proved it as one of stable reference genes^18,42–44^. Although GAPDH has been referred as a suitable gene for efficient normalization in fish^18,42–45^, it was found to be having medium stability. Similarly, GAPDH was found to be unstable while studying validation of reference genes in silverside, *Odontesthes humensis* under varied environmental conditions^37^. Further, the algorithms used in this study contradicts use of β-actin as reference gene in this fish species, particularly while using skin tissue because of medium stability. However, it does not mean the previous studies using β-actin as reference should not be disregarded, rather emphasis needs to be given in future in selecting reference genes. Similarly, AT gene has been described of having high stability in validation of reference genes in fish experiments in earlier studies both under normal and infection cases^33,34^. However, in the present study it was found to be least stable one like evident in silversides^37^.

To demonstrate the influence of reference genes for normalisation the most stable (ARP0 and EF1α) and the least stable (RPo and AT) genes were utilized while studying the expression of both innate and specific immune related genes. The findings obtained confirmed the significant difference in transcript pattern of target immune genes when two most stable and two least stable genes were used to normalise the transcript data. Hence, it was concluded that choosing inapt reference gene without validation could lead to misinterpretation of obtained results from experiments. Hence, the normalised fold expressions obtained using the most stable genes i.e. ARP0/EF1α was further interpreted here while describing the expression pattern of immune related genes with regard to parasitic infection in *L. rohita*.

Skin primarily serves as the first line of defense in all vertebrates. It is the primary target site during ectoparasitic infection, local or systemic inflammations and serve as superintend to susceptibility or resistance to any infection^22,46^. A large number of immune-related molecules are directly or indirectly involved in this inflammatory response. Hence, the present study inspected different immune related genes contemporary in *L. rohita* skin during parasitic infection (*A. siamensis*). Toll-like receptors (TLRs) are type I transmembrane innate immune receptors that recognize pathogens or their molecular signals and activate signaling cascades to induce early non-specific immune response in the host body. Among various types of TLRs, TLR22 is solely present in teleosts and amphibians, plays a distinctive role in innate immunity. The participatory role of TLR22 during lice infection in *Catla catla* has been reported earlier^47^. Further, TLR22 was found to be very highly up-regulated in mucus of rohu which declined as the infection progressed^48^. Interestingly, in the present study TLR22 expression was noticed to be stable in skin during *A. siamensis* infection up to a period of 20 dpi and then up-regulated during 30 dpi. Kar et al.^9^ also mentioned the upregulation of TLR22 is directly co-related with severity of *A. siamensis* infection. Hence, the probable reason behind delayed expression of TLR22 is correlated with the severity of the infection which was much more evident here on day 30 or a stage where the tissue damage-induced signal is being adequately recognized as a ligand for TLR recognition. However, the role of other TLRs in this recognition mechanism may be looked into.

Although three classes of Igs (IgM, IgD, and IgT/Z) have been detected in teleosts, IgM and IgD are commonly reported from fish to most vertebrate classes, thus hypothesizing as ancestral isotypes. *Argulus* infection leads to down-regulation in IgM expression and up-regulation of IgZ expression in skin tissue of juvenile rohu^10^. In another study, an early up-regulation of IgD in skin and mucus samples was noticed during argulosis^9^. Surprisingly, in the present study IgD expression was found unchanged when ARP0 was used as housekeeping gene and down-regulated when EF1α used as reference gene. Interestingly, only a transient early up regulation of IgD was observed when RPo and AT used as reference genes. Hence, it proves how crucial it is to determine a suitable reference gene before going into the gene expression study.

Interleukin-10 (IL-10) is a pleiotropic cytokine synthesis inhibitory factor being expressed in various fish species and higher vertebrates. It has been reported as anti-inflammatory cytokine and activated during infection. It limits the production of excess inflammatory molecules during a parasitic infection by keeping the immune reaction under optimum condition^41^. In the present study IL-10 expression was found up-regulated 10 dpi onwards using ARP0 as a reference gene. The increase in expression of IL-10 during the infection process might be playing a role to maintain an appropriate balance of inflammatory response. Interestingly, its expression was not much influenced when other reference genes being used.

Major histocompatibility complex (MHC) molecules are important components of the adaptive immune system responsible for the activation of adaptive immunity in the host. In the present study, gradual decrease in expression of MHCII and transient increase in expression of MHCI at later time point suggested the involvement of CTL response rather than Th response in the inflammatory process caused by this parasite. Up-regulated expression of ApoA-I in skin, mucus, liver and anterior kidney of rohu infected with *A. siamensis* has been noticed earlier^39^. In the present study also the expression of ApoA-I showed upregulation at 10 dpi onward. Paul et al.^41^ also described prominent role of antimicrobial peptides in parasite pathogenesis. Since ApoA-I has anti-inflammatory and anti-apoptic role, its increased expression during skin parasite infection needs to be further explored.

## Conclusions

From seven selected candidate reference genes, we found that ARP0 and EF1α as most stable genes with high level of basal expression and low variability during *A. siamensis* infection in rohu using skin tissue in RT-qPCR expression analysis. RPo and AT showed high variability with low level of expression and considered as least stable genes for expression analysis. It seems to be the first study in this fish species where reference genes are being validated using skin tissue both in normal and followed by ectoparasitic infection. ARP0 has hardly being used as reference gene in fish species although this study proved to be a very stable gene for expression analysis. Hence, future studies in other fish species in different experimental conditions are needed to prove it as a reference gene candidate. Validation of candidate reference genes were carried out by looking expression of immune genes viz., MHC II, MHC I, IL 10, TLR 22, IgD and ApoA-I by using two stable and two least stable genes as housekeeping genes in *Argulus*-infected rohu skin at different time points of infection. An increased expression of immune genes TLR 22, IL 10, MHC I and ApoA-I were found at variable time points of infection. MHC II and IgD was found to be down-regulated or unchanged during the post-infection period indicating the role of inflammation and immune modulation process at the site of attachment of parasites in the host to render protection.

## Supplementary Information

Supplementary Figure 1.

Supplementary Figure 2.

Supplementary Information 3.

## Data Availability

All data generated or analysed during this study are included in this published article (and its Supplementary Information files).
