# Supplementary figures and images for: Stability evaluation and validation of appropriate reference genes for real-time PCR expression analysis of immune genes in the rohu (Labeo rohita) skin following argulosis
Source: Sci Rep. 2023 Feb 15;13:2660. doi: 10.1038/s41598-023-29325-1 (PMC9932016; doi:10.1038/s41598-023-29325-1)

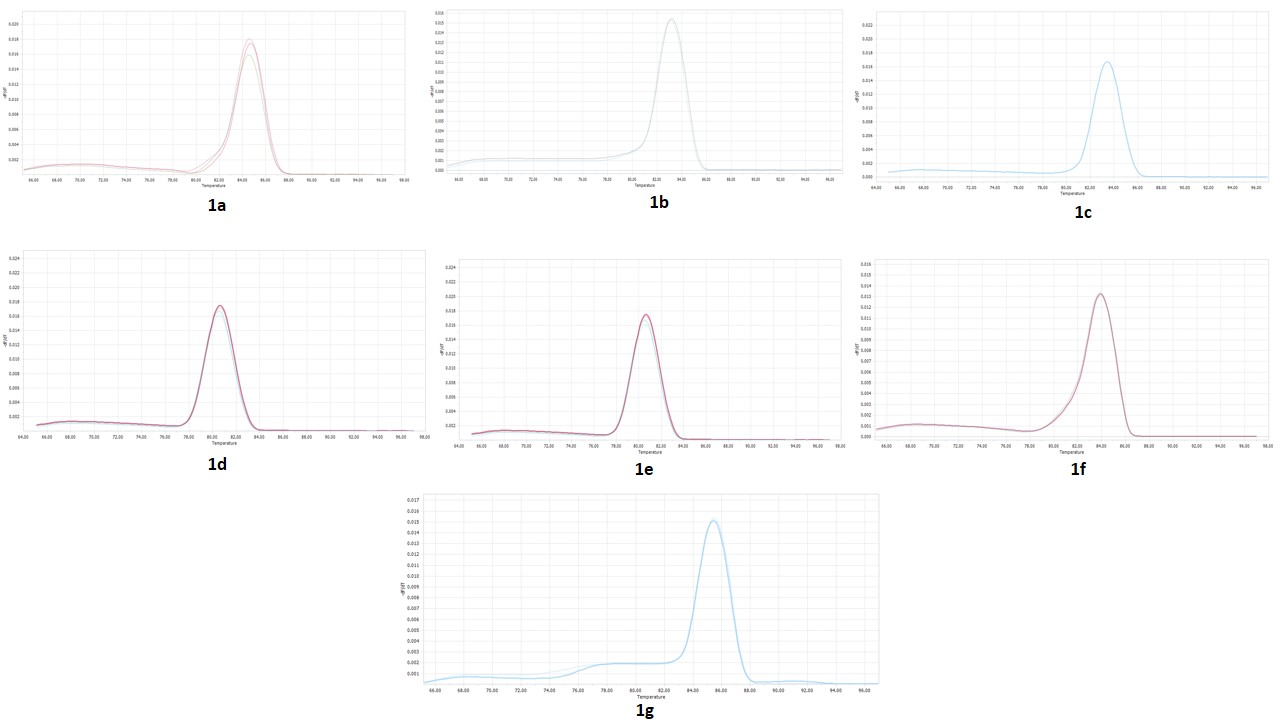

Supplement: Supplementary file 1 — Supplementary Figure 1. [file 41598_2023_29325_MOESM1_ESM.jpg]

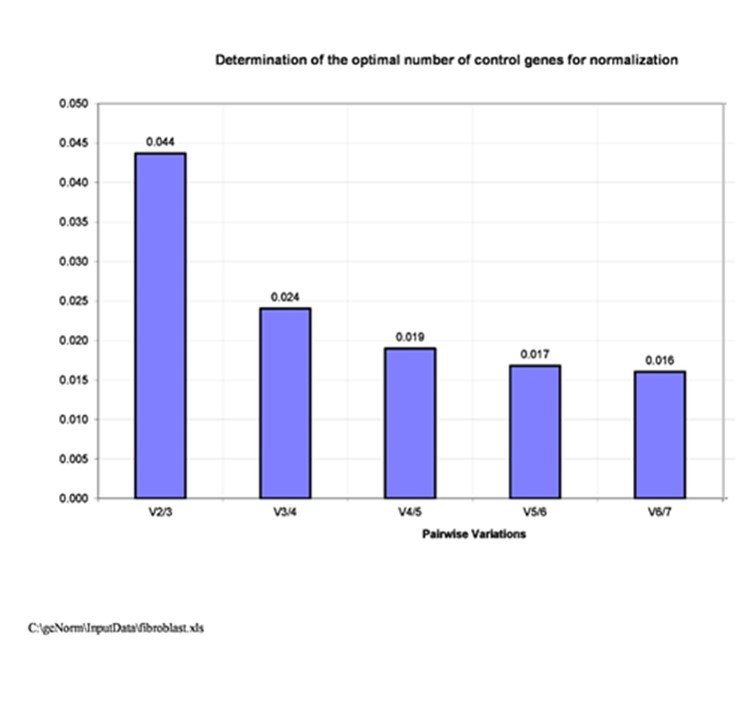

Supplement: Supplementary file 2 — Supplementary Figure 2. [file 41598_2023_29325_MOESM2_ESM.jpg]
