# Supplementary material for: Stability evaluation and validation of appropriate reference genes for real-time PCR expression analysis of immune genes in the rohu (Labeo rohita) skin following argulosis
Source: Sci Rep. 2023 Feb 15;13:2660. doi: 10.1038/s41598-023-29325-1 (PMC9932016; doi:10.1038/s41598-023-29325-1)
Supplement: Supplementary file 3 — Supplementary Information 3. [file 41598_2023_29325_MOESM3_ESM.docx]

**Legend to the supplementary Figures**

Suppl. Fig. 1. Melt curve of selected reference genes (1a) ARP0, (1b) AT, (1c) β actin, (1d) EF1α, (1e) GAPDH, (1f) RPo and (1g) RL 10

Suppl. Fig. 2. Pairwise variation values of geNorm analysis

Supplementary table 1a. R value of BestKeeper and M value of geNorm analysis

| **Gene name** | **BestKeeper Pearson correlation coefficient (R)** | **Genorm Stability value (M)** |
| --- | --- | --- |
| ARP0 | 0.830 | 2.057 |
| β-actin | 0.796 | 2.321 |
| EF1α | 0.933 | 1.787 |
| GAPDH | 0.903 | 1.435 |
| RL 10 | 0.950 | 1.435 |
| RPo | 0.766 | 5.507 |
| AT | 0.971 | 4.262 |

Supplementary table 1b. Intergroup and intragroup variation value by NormFinder analysis

| **Gene name** | **Intergroup variation** | | | | | | **Intragroup variation** | | | | | |
| --- | --- | --- | --- | --- | --- | --- | --- | --- | --- | --- | --- | --- |
|  | **0 dpi** | **1 dpi** | **5 dpi** | **10 dpi** | **20 dpi** | **30 dpi** | **0 dpi** | **1 dpi** | **5 dpi** | **10 dpi** | **20 dpi** | **30 dpi** |
| ARP0 | -1.656 | -2.289 | 0.846 | 1.548 | 1.307 | 0.244 | 0.011 | 0.108 | 0.003 | 0.070 | 0.066 | 2.093 |
| β-actin | 0.066 | 2.999 | -1.137 | -0.783 | -0.848 | -0.297 | 1.855 | 6.588 | 0.115 | 0.412 | 0.030 | 2.642 |
| EF1α | 0.884 | -0.560 | -0.569 | 0.153 | -1.240 | 1.331 | 2.610 | 1.790 | 0.119 | 3.489 | 0.137 | 0.134 |
| GAPDH | -1.133 | -0.979 | 0.821 | -0.339 | 1.236 | 0.393 | 0.006 | 2.781 | 0.003 | 0.486 | 0.799 | 4.274 |
| RL 10 | -1.006 | -1.594 | -0.069 | 0.395 | 0.483 | 1.791 | 0.270 | 0.269 | 0.522 | 0.005 | 0.411 | 0.688 |
| RPo | 2.789 | 2.100 | -0.491 | -0.219 | -0.181 | -3.998 | 0.248 | 0.062 | 0.004 | 0.385 | 2.227 | 35.921 |

Supplementary table 1c.The fold expression, standard deviation and standard error values of TLR 22 against the housekeeping genes, ARP0, EF1α, RPo and AT

| Time period | Housekeeping gene | Fold expression | Standard error | Statistics analysis |
| --- | --- | --- | --- | --- |
| 0 day  1 day  5 day  10 day  20 day  30 day | ARP0  ARP0  ARP0  ARP0  ARP0  ARP0 | 0.996863473  0.037702187  0.303553161  11.02795022  1.224463545  41.56496007 | 0.017949825  0.010363336  0.060033334  9.532105515  0.698591544  13.3245878 | a  a  a  a  a  b |
| 0 day  1 day  5 day  10 day  20 day  30 day | EF1α  EF1α  EF1α  EF1α  EF1α  EF1α | 2.219935601  0.082204141  0.099709766  0.04374516  0.852866479  543.5533 | 1.628944623  0.026595552  0.006176016  11.58765599  0.007976627  146.1096683 | a  a  a  a  a  b |
| 0 day  1 day  5 day  10 day  20 day  30 day | RPo  RPo  RPo  RPo  RPo  RPo | 1.335339575  1.21662176  0.205160786  0.13277961  0.475137833  1.070241047 | 0.737287707  1.030498162  0.096761276  0.078187278  0.268199097  0.615444045 | a  a  a  a  a  a |
| 0 day  1 day  5 day  10 day  20 day  30 day | AT  AT  AT  AT  AT  AT | 1.005274535  0.513210381  0.133133729  0.049619563  0.008946594  1.107535247 | 0.074465484  0.410159573  0.119864228  0.030294916  0.000555203  0.745070896 | a  a  a  a  a  a |

Supplementary table 2. The fold expression, standard deviation and standard error values of IgD against the housekeeping genes, ARP0, EF1α, RPo and AT

| Time period | Housekeeping gene | Fold expression | Standard error | Statistics analysis |
| --- | --- | --- | --- | --- |
| 0 day  1 day  5 day  10 day  20 day  30 day | ARP0  ARP0  ARP0  ARP0  ARP0  ARP0 | 3.386249651  0.849108556  0.236660047  0.049152266  0.516988432  0.7313431 | 3.032051289  0.648405951  0.151659739  0.021117373  0.28072142  0.35046315 | a  a  a  a  a  a |
| 0 day  1 day  5 day  10 day  20 day  30 day | EF1α  EF1α  EF1α  EF1α  EF1α  EF1α | 2.007722878  0.575850894  0.005078209  0.063438494  0.005994612  0.174358897 | 1.03238  0.43044  0.002908  0.056782  0.002769  0.105125 | b  a  a  a  a  a |
| 0 day  1 day  5 day  10 day  20 day  30 day | RPo  RPo  RPo  RPo  RPo  RPo | 24.9451  141.0204  4.334524  0.896652  1.334645  0.049266 | 11.714  66.52495  0.437985  0.389169  0.366117  0.010066 | a  b  a  a  a  a |
| 0 day  1 day  5 day  10 day  20 day  30 day | AT  AT  AT  AT  AT  AT | 5.290013  24.27796  0.247395  1.365396  6.964781  0.011057 | 4.442654747  5.270786427  0.155214164  0.592615634  5.643840485  0.003986175 | a  b  a  a  a  a |

Supplementary table 3. The fold expression, standard deviation and standard error values of IL 10 against the housekeeping genes,ARP0, EF1α, RPo and AT

| Time period | Housekeeping gene | Fold expression | Standard error | Statistics analysis |
| --- | --- | --- | --- | --- |
| 0 day  1 day  5 day  10 day  20 day  30 day | ARP0  ARP0  ARP0  ARP0  ARP0  ARP0 | 0.507418247  1.871625055  26.71580989  88.11330285  16.7176788  177.2317149 | 0.021090773  1.651987027  1.195570566  34.98244888  1.720868581  112.7865597 | a  a  a  b  a  b |
| 0 day  1 day  5 day  10 day  20 day  30 day | EF1α  EF1α  EF1α  EF1α  EF1α  EF1α | 0.33238592  0.01876077  0.49966856  4.907554438  0.176259824  0.220831992 | 0.146824167  0.01222804  0.265603685  2.659334508  0.107021131  0.118997938 | a  a  a  b  a  a |
| 0 day  1 day  5 day  10 day  20 day  30 day | RPo  RPo  RPo  RPo  RPo  RPo | 2.206283475  9.854474241  0.098625096  0.03723769  0.071884884  0.002715866 | 1.474857581  9.011959577  0.065281691  0.018171652  0.056542666  0.002389194 | a  a  a  a  a  a |
| 0 day  1 day  5 day  10 day  20 day  30 day | AT  AT  AT  AT  AT  AT | 1.441420531  1.016048471  5.230656821  5.376554385  6.36085055  1.420764602 | 0.381648785  0.114399885  0.429136286  2.170360797  2.33302835  0.321657253 | a  a  b  b  b  a |

Supplementary table 4. The fold expression, standard deviation and standard error values of MHC II against the housekeeping genes, ARP0, EF1α, RPo and AT

4.081066561 0.213701584 2.606859468 0.854170209 0.306193468 0.241899139

| Time period | Housekeeping gene | Fold expression | Standard error | Statistics analysis |
| --- | --- | --- | --- | --- |
| 0 day  1 day  5 day  10 day  20 day  30 day | ARP0  ARP0  ARP0  ARP0  ARP0  ARP0 | 1.204171982  0.18660724  0.096927199  0.131714661  0.140139243  0.011853977 | 0.415298631  0.107491777  0.00653121  0.072989196  0.073000871  0.00653121 | b  a  a  a  a  a |
| 0 day  1 day  5 day  10 day  20 day  30 day | EF1α  EF1α  EF1α  EF1α  EF1α  EF1α | 4.392771252  0.647953735  6.471755861  0.876389929  0.340563825  0.366190001 | 4.081066561  0.213701584  2.606859468  0.854170209  0.306193468  0.241899139 | a  a  a  a  a  a |
| 0 day  1 day  5 day  10 day  20 day  30 day | RPo  RPo  RPo  RPo  RPo  RPo | 1.481281217  3.344181792  2.12322072  1.088842425  1.26677127  0.002803494 | 0.86668382  1.196756303  1.326269385  0.243461784  0.553672417  0.001188102 | ab  b  ab  ab  ab  a |
| 0 day  1 day  5 day  10 day  20 day  30 day | AT  AT  AT  AT  AT  AT | 3.842805426  0.538020819  1.338320768  0.145744868  0.061962787  0.016834278 | 2.859992758  0.265668288  1.289713557  0.096871415  0.023071961  0.006706974 | a  a  a  a  a  a |

Supplementary table 5. The fold expression, standard deviation and standard error values of MHC I against the housekeeping genes, ARP0, EF1α, RPo and AT

| Time period | Housekeeping gene | Fold expression | Standard error | Statistics analysis |
| --- | --- | --- | --- | --- |
| 0 day  1 day  5 day  10 day  20 day  30 day | ARP0  ARP0  ARP0  ARP0  ARP0  ARP0 | 0.531897326  0.091444  0.309451216  22.46717504  1.743252416  0.032816096 | 0.195088932  0.028240318  0.018509185  5.292792428  1.102160624  0.006915346 | a  a  a  b  a  a |
| 0 day  1 day  5 day  10 day  20 day  30 day | EF1α  EF1α  EF1α  EF1α  EF1α  EF1α | 0.258114227  0.170779455  0.258114227  71.49377044  0.274991087  0.025094663 | 0.062353935  0.073847821  0.04409089  17.44231669  0.151788638  0.008725359 | a  a  a  b  a  a |
| 0 day  1 day  5 day  10 day  20 day  30 day | RPo  RPo  RPo  RPo  RPo  RPo | 0.549967996  0.104642644  1.138965256  2.097334931  0.137677457  0.268478682 | 0.096208419  0.022768408  0.355805463  0.778929528  0.10570978  0.052542949 | a  a  ab  b  a  a |
| 0 day  1 day  5 day  10 day  20 day  30 day | AT  AT  AT  AT  AT  AT | 0.328895619  0.036353399  1.317385103  1.977615538  1.926615759  0.010893253 | 0.010255463  0.016464041  0.988507216  1.504586715  0.726254263  0.000707484 | a  a  a  a  a  a |

Supplementary table 6. The fold expression, standard deviation and standard error values of ApoA-I against the housekeeping genes, ARP0, EF1α, RPo and AT

| Time period | Housekeeping gene | Fold expression | Standard error | Statistics analysis |
| --- | --- | --- | --- | --- |
| 0 day  1 day  5 day  10 day  20 day  30 day | ARP0  ARP0  ARP0  ARP0  ARP0  ARP0 | 0.276428505  0.028889096  0.413196888  0.521708646  1.114385518  0.914869675 | 0.088221028  0.000982266  0.329684002  0.191060357  0.192570589  0.205578611 | ab  a  ab  abc  c  bc |
| 0 day  1 day  5 day  10 day  20 day  30 day | EF1α  EF1α  EF1α  EF1α  EF1α  EF1α | 0.300770219  0.005673629  1.07745313  1.463719476  0.018155272  2.528606004 | 0.04768929  0.001142052  0.318654521  1.426749649  0.01317896  1.591333775 | a  a  a  a  a  a |
| 0 day  1 day  5 day  10 day  20 day  30 day | RPo  RPo  RPo  RPo  RPo  RPo | 1.029147322  0.446515261  0.003481945  0.024789827  0.051614501  0.030974582 | 0.155948717  0.351730471  8.13573E-05  0.01786487  0.031518663  0.014531633 | b  a  a  a  a  a |
| 0 day  1 day  5 day  10 day  20 day  30 day | AT  AT  AT  AT  AT  AT | 1.073036221  1.070809792  0.125048319  1.346510055  0.612634742  2.118905031 | 0.252672266  0.531559565  0.052140548  0.97036689  0.33704243  0.245971207 | ab  ab  a  ab  ab  b |
